# Oncogenic K-Ras and Loss of Smad4 Mediate Invasion by Activating an EGFR/NF-κB Axis That Induces Expression of MMP9 and uPA in Human Pancreas Progenitor Cells

**DOI:** 10.1371/journal.pone.0082282

**Published:** 2013-12-05

**Authors:** Alakesh Bera, Shujie Zhao, Lin Cao, Paul J. Chiao, James W. Freeman

**Affiliations:** 1 Department of Medicine, Division of Medical Oncology, University of Texas Health Science Center at San Antonio, San Antonio, Texas, United States of America; 2 Cancer Therapy and Research Center, Experimental and Developmental Therapeutics Program, San Antonio, Texas, United States of America; 3 Research and Development, Audie Murphy Veterans Administration Hospital, San Antonio, Texas, United States of America; 4 Department of Molecular and Cellular Oncology, The University of Texas MD Anderson Cancer Center, Houston, Texas, United States of America; Technische Universität München, Germany

## Abstract

Activating K-Ras mutations and inactivating mutations of Smad4 are two common genetic alterations that occur in the development and progression of pancreatic ductal adenocarcinomas (PDAC). To further study the individual and combinatorial roles of these two mutations in the pathogenesis of PDAC, immortalized human pancreas nestin postive cells (HPNE) were genetically modified by either expressing oncogenic K-Ras (HPNE/K-Ras), by shRNA knock down of Smad4 (HPNE/ShSmad4) or by creating both alterations in the same cell line (HPNE/K-Ras/ShSmad4). We previously found that expression of oncogenic K-Ras caused an increase in expression of EGFR and loss of Smad4 further enhanced the up regulation in expression of EGFR and that this increase in EGFR was sufficient to induce invasion. Here we further investigated the mechanism that links mutational alterations and EGFR expression with invasion. The increase in EGFR signaling was associated with up regulation of MMP9 and uPA protein and activity. Moreover, the increase in EGFR signaling promoted a nuclear translocation and binding of RelA (p65), a subunit of NF-κB, to the promoters of both MMP-9 and uPA. Treatment of HPNE/K-Ras/ShSmad4 cells with an inhibitor of EGFR reduced EGF-mediated NF-κB nuclear translocation and inhibitors of either EGFR or NF-κB reduced the increase in MMP-9 or uPA expression. In conclusion, this study provides the mechanism of how a combination of oncogenic K-Ras and loss of Smad4 causes invasion and provides the basis for new strategies to inhibit metastases.

## Introduction

The prognosis of patients with pancreatic cancer is extremely poor with a five-year survival rate of less than 5% [1] [2]. Approximately 90% of these cancers are believed to arise from ductal epithelial cells and are classified as PDACs [3]. Two common genetic alterations that occur in the development and progression of PDAC are activating mutation of *K-Ras* and inactivation of *DPC4* (codes for tumor suppressor protein Smad4) by allelic deletion or intragenic mutations. Mutation of *K-Ras* acts as an initiating event in development of PADC; whereas, alterations of *Smad4* occur during progression of the disease [4,5] [6].  Smad4 is necessary for canonical TGF-β /Smad signaling and mutation of *Smad4* is a major mechanism causing loss of TGF-β tumor suppressor activity. 

PDAC is aggressive and highly metastatic in nature [7] [8]. The metastatic process involves intravasation and extravasation of tumor cells, followed by reimplantation of tumor cells, formation of a new tumor stroma, and neoangiogenesis to consolidate a secondary tumor at a distant site [9] [3]. Cell migration and invasion involve a number of interdependent processes including the formation of cell protrusions or lamellipodia in the direction of movement, retraction of posterior formations, and establishment and rupture of adhesive contacts (focal contacts) between the cell and the extracellular matrix (ECM). Focal contacts may be broken by extracellular proteolytic enzymes, such as serine proteases of the plasmin system or matrix metalloproteinases (MMPs), thereby enabling cell migration [3]. Degradation of the extracellular matrix and components of the basement membrane by proteases facilitates the detachment of tumor cells, facilitates their crossing of tissue boundaries, and invasion into adjacent tissue compartments. In recent years, the importance of tumor-associated proteases in invasion and metastasis has been demonstrated for a variety of solid malignant tumors. The serine protease uPA, its inhibitor PAI-1, and the MMPs play important roles in these processes [10] [2]. 

Urokinase plasminogen activator (uPA) facilitates extracellular matrix degradation by converting zymogen plasminogen into plasmin, a serine protease with broad substrate specificity [4]. It binds to a highly glycosylated receptor (uPAR, CD87) that focuses the proteolytic activity to the cell surface. The uPA inhibitor PAI-1 regulates uPA activity and plays an important role in re-implantation of disseminated tumor cells and formation of a new tumor stroma at the site of the metastasis. Besides its role in proteolysis, the plasminogen activation system stimulates cell proliferation and modulates cell adhesion [11,12].

The MMP family of proteases has also been implicated in tumor cell invasion and metastasis. MMPs are characterized by a zinc co-ordinate active site and are classified according to homologies in sequence and substrate affinity [3]. The two metalloproteinases, MMP-2 and MMP-9 (72 kDa and 92 kDa type IV collagenases or gelatinase A and gelatinase B, respectively), have been associated with the malignant phenotype by their unique ability to degrade type IV collagen, which is a major component of the basement membrane [9] . Collectively, MMPs are able to degrade all components of the ECM. They play key roles in normal physiologic processes involving ECM remodeling, such as wound healing, angiogenesis, and development. MMPs also participate in inﬂammation, tumor invasion, and metastasis [8] [10]. Among these MMPs, MMP-9 has been particularly involved in the migration of several cell types, i.e., macrophages, T lymphocytes, and eosinophils, through reconstituted membrane. Induction of keratinocyte migration by EGF and HGF has also been found to coincide with the induction of MMP-9 activity [13]. 

The phosphotyrosine kinase receptors EGFR and erbB2 are known to be up regulated concomitant with expression of oncogenic K-Ras [14]. The current study was undertaken to determine whether up regulation of EGFR caused by K-Ras and loss of Smad4 play role in the increase invasion seen in these cells. To examine this possibility, primary human cells derived from the ducts of the pancreas and immortalized with hTERT were used. This immortalized cell line model was previously described and is positive for the expression of nestin, is diploid and expresses wild type p16^Ink4a^, p53 and K-Ras and is referred to as human pancreas nestin expressing cells or HPNE [15] [16]. To block Ras-induced senescence and allow oncogenic Ras expression, these cells were modified to produce the HPV16, E6 and E7 proteins. From these E6/E7 cells (thereafter referred as HPNE), isogenic matched cell lines were generated by expressing K-Ras ^(G12D)^ [17] and/or by knocking down Smad4. Expressing K-Ras^(G12D)^ was sufficient to substantially up regulate the expression of both EGFR and erbB2 but not Ron kinase or IGF-1R [16].

A biologic link between NF-κB and EGFR activities was suggested by demonstrating that increased EGFR signaling induces NF-κB activation [18] [19] [20]. NF-κB expression can be constitutively activated in many cancer types as well as NF-κB expression is also activated in response to different stimuli, including chemotherapy [21] [22]. This led to the development of inhibitors to NF-κB pathway as a potential strategy for cancer therapy. Further, NF-κB is activated constitutively in human pancreatic cancer cell lines but not in normal pancreatic tissues suggesting that NF-κB plays a crucial role in pancreatic cancer progression. On the basis of these findings, other preclinical studies examined the possibility of restoring apoptosis treating cancer cells with an anti-EGFR antibody that consequently decreased NF-κB activity [23,24]. 

This study examined the biologic consequence of two molecular alterations that are commonly found in PDAC. These alterations are gain of oncogenic K-Ras and loss of Smad4 that prevents canonical TGF-β signaling. The results indicate that oncogenic Ras and loss of Smad4 signaling cooperate to up-regulate EGFR/ NF-κB axis, which plays a role in promoting invasion through induced expression of the matrix proteinases MMP9 and uPA.

## Materials and Methods

### Cell lines and reagents

The hTERT-immortalized human pancreas nestin expressing cell line (hTERT-HPNE) modified to express E6/E7 alone (refer therein as HPNE) or E6/E7 in conjunction with oncogenic K-Ras (designated as HPNE/K-Ras) were obtained from Michel M. Ouellette (University of NebRaska Medical Center, Omaha, NebRaska) [15]. From these two cell lines, we generated the isogenic matched cell lines in which Smad4 is silenced by infecting these cells with a pSuper/Smad4 shRNA plasmid. A stable pool was selected by the limited dilution and knock down of Smad4 expression was confirmed by Western blot. These cell lines were designated as HPNE/ShSmad4 or HPNE/K-Ras/ShSmad4, respectively [16]. All the cell lines were maintained in medium M3/D [3 parts DMEM (Invitrogen, Grand Island, NY) and 1 part M3F medium (INCELL Corporation LLC, San Antonio, TX) supplemented with 5% FBS [15]. The human recombinant EGF was purchased from R&D Systems (Minneapolis, MN). The EGFR inhibitor, AG1478 was purchased from Calbiochem (San Diego, CA). NF-κB inhibitor Bay11-7082 and and PI3K inhibitor Ly294002 were bought from Sigma-Aldrich and Calbiochem respectively. 

### Western blots analyses and immuno-fluorescence staining

 Western blot analysis was performed as described previously [16]. Primary antibodies used were as follows: Cyclin D2, K-Ras, uPA NF-κB (p65) and actin were from Santa Cruz (Santa Cruz, CA); anti-EGFR and anti-phos-EGFR^Tyr1068^, and MMP9 were purchased from Cell Signaling Technology (Beverly, MA). Horseradish peroxidase-conjugated secondary antibodies were purchased from Amersham Biosciences (Piscataway, NJ). 

 For immuno-fluorescence staining (IF), cells grown on round cover-slips into a 24-well plate. The cells were fixed in 4% paraformaldehyde for 15 minutes at room temperature and permeabilized with 0.1% Triton X-100. Non-specific sites were blocked with 5% goat serum. The primary antibody to EGFR was obtained from Santa Cruz and Alexa Fluor-488 goat anti-rabbit IgG was purchased from Molecular Probes (Life Tech., CA). MMP9 and uPA primary antibodies were also used for the staining of respective proteins. Cell nuclei were stained with DAPI for 5 min and the slides were then mounted with PremaFluo aqueous mountant (Immuno Thermo, Pittsburgh, PA). Fluorescence images were captured with an Olympus FV1000 scanning confocal microscope furnished with a 60 x objectives and Olympus FV1000 software. The processing of the captured images was performed by *Image J* (NIH) photo imaging software. 

### Matrigel Invasion Assays

 The invasive behavior of cells was analyzed by Matrigel invasion assays as described previously [25]. Briefly, 3 × 10^4^ cells/well were placed in a 24-well Matrigel invasion chambers (Becton Dickinson Labware, Franklin Lakes, NJ) in 0.5 ml of serum free medium M3/D. The outer chambers contained 0.7 ml of medium m3/D supplemented with 5% FBS. After 24 hours of incubation the cells on the top surface of the membrane were gently removed with cotton swaps. The cells migrating to the undersurface of the membrane were fixed in 70% ethanol and stained with crystal violet. The invasion values were determined by calculating the percentage of fraction occupied by the invaded cells after taking the image of undersurface. The analysis was done by *Image J* software using ‘analyze particles’ option.

### Gelatin Zymography

 Matrix metalloproteinase MMP9 enzymatic activity in serum free medium was determined by SDS-PAGE gelatin zymography. HPNE and the other related cell lines were cultured in the recommended M3/D media supplemented with 5% FBS and antibiotics in 6-well tissue culture plates. At near confluence, the cells were washed with PBS and then 1 ml of serum free medium was added. The cultures were incubated and treated with EGF (50 ng/ml) or inhibitors (AG1478, 10 μM, Bay11-7982, 10 μM and Ly294002, 10 μM) or combination of EGF and inhibitors. After 24 hours of incubation the media were collected and MMP9 activity was assayed by gelatin zymography. Sample proteins were resolved by electrophoresis under non-denaturing conditions in 8% polyacrylamide–SDS gel containing 1 mg/ml porcine skin gelatin (Sigma Chemical Co., St. Louis, MO). After electrophoresis, the gels were washed twice for 30 min in 2.5% (v/v) Triton X-100 to remove SDS and then incubated overnight at 37°C in 50 mM Tris–HCl, pH 7.6, containing 5 mM CaCl_2_. After staining with 0.5 % Coomassie brilliant blue R-250, gelatin degrading proteins were recognized as clear zones of lysis against a blue background. 

### Casein Zymography

 Casein Zymography assay previously described by Marshall et al. [26] was used to determine the types and molecular weights of plasminogen activators. Culture media were collected as above. Samples were resolved under the same conditions as gelatin zymography except that the gel contained 2 mg/ml bovine α-casein (Sigma) and 0.5U/ml plasminogen (Calbiochem) instead of gelatin. Gels were incubated overnight at 37°C in 100 mM glycine, 10 mM EDTA (in order to inhibit MMPs), pH 8.3. uPA (0.01U) prepared from human urine (672112, Calbiochem) was used as a positive control.

### Chromatin Immunoprecipitation (ChIP) Assay

 Tow different cells (HPNE and HPNE/K-Ras/ShSmad4) were taken in order to examine the promoter binding phenomenon of NF-κB towards MMP9 and uPA promoter regions. Cells were induced with EGF (50 ng/ml) for one hour in order to better nuclear translocation of NF-κB. ChIP assay was carried out using ChIP-IT® Express Chromatin Immunoprecipitation Kits (Active Motif, Carlsbad, CA) following manufacturer's protocol. In brief, freshly prepared 18.5% formaldehyde was added to the cells (~1×10^7^ cells in 100 mm dish) at a final concentration of 1%. Cells were incubated at 37°C for 10 min, and then excess formaldehyde was quenched by addition of 5M glycine. After washing twice, the cells were scraped into 2 ml cold PBS containing 1× protease inhibitor cocktail II. The cells were pelleted and then resuspended in Cell Lysis Buffer containing 1× protease inhibitor cocktail II. Nuclei were isolated after Dounce homogenization and resuspended in Nuclear Lysis Buffer containing 1 X protease inhibitor cocktail II. The samples were sonicated on ice using a Bioruptor sonicator (Diagenode Inc., Sparta, NJ) to shear the cross-linked DNA to an average length of 200–1000 bp and centrifuged at 13,000 rpm to remove insoluble material. The chromatin solutions were diluted 10-folds using dilution buffer, and 10 µl of each was reserved as total input control. Diluted chromatin solutions were precleared with salmon sperm DNA-protein A magnetic beads for 1 hr, and then incubated with protein G magnetic beads (Dynabeads Protein G, Life technologies, CA) and antibodies specific for EGFR and NF-κB (p65) (Santa Crutz, CA) or nonspecific IgG overnight at 4°C. The immunoprecipitated complex-magnetic beads were collected using magnetic separator and washed according to manufacturer's instruction. The pellets were then incubated with proteinase K in ChIP Elution buffer for 2.5 hrs at 65°C with shaking to elute immunocomplex and reverse cross-link. The samples were incubated at 95°C for 10 min and DNA was purified according to manufacturer's instruction. 1 µl of each of the purified DNA was used as template for 30 cycles of PCR amplification using designated primers. The PCR products were then analyzed by agarose gel electrophoresis and visualized using EB staining. PCR ampliﬁcation were performed with the forward (5’-TGTCCCTTTACTGCCCTGA-3’) and reverse (5’-ACTCCAGGCTCTGTCCTCCTCTT-3’) primers, which were speciﬁcally designed from the MMP-9 promoter region (-657 to -484) [27]. For uPA promoter amplification Set 2 primers (forward primer 5’-ACTCTCCCTGCCTTCCTTCT-3’ and reverse primer 5’-GTGATTCTGTCACCCCCATC-3’ uPA promoter were used as described in Shimizu et al (2011) [28].

### Statistical analysis

The Student’s unpaired t-test was used to compare individual group means. A p value of <0.05 was considered as statistically significant. All values in the figures and text were expressed as the mean ± S.D.

## Results

### Oncogenic K-Ras expression and loss of Smad4 cooperate to induce EGFR expression and MMP9 and uPA expression and activity

**Figure 1:**
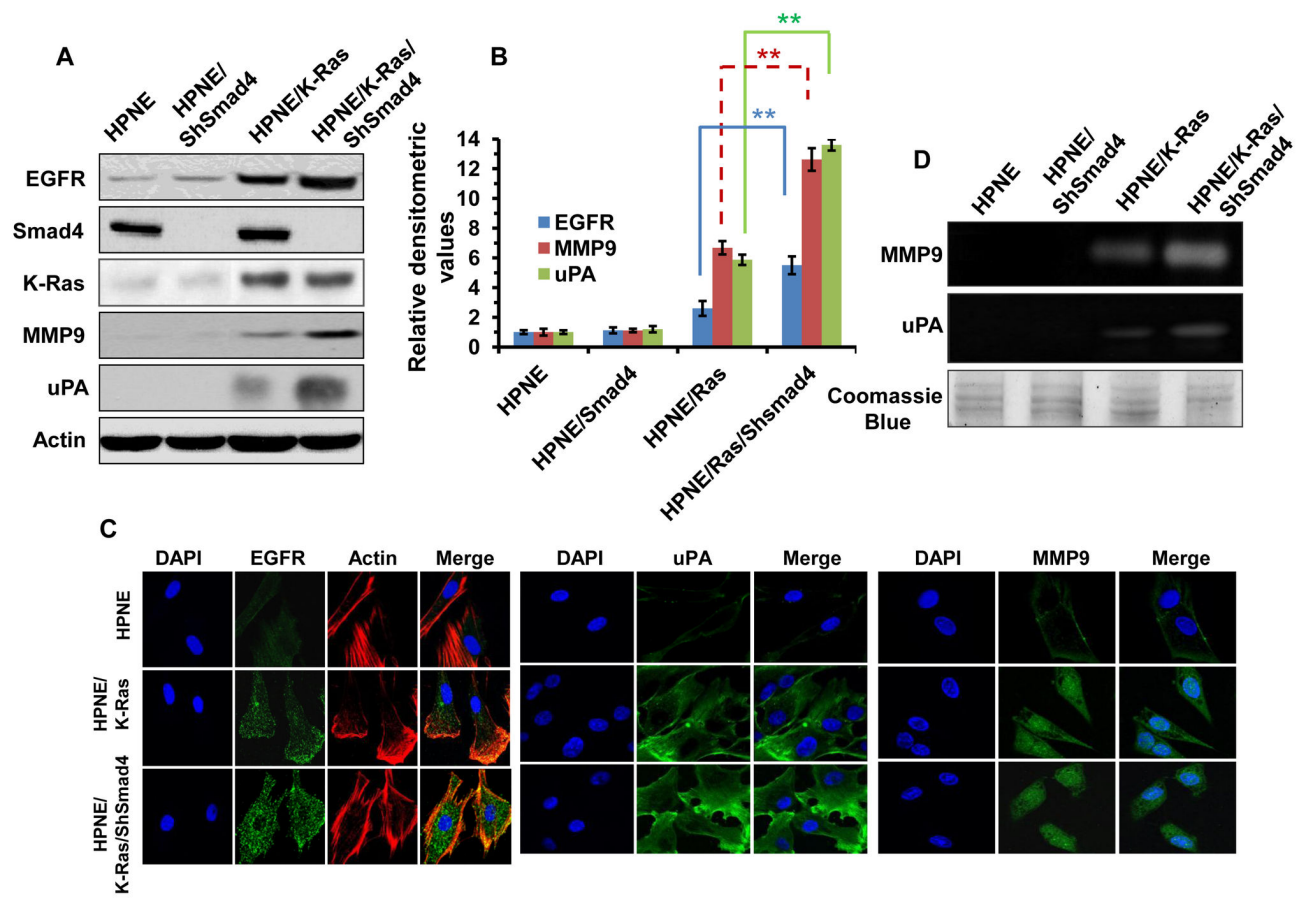
Characterization of genetically modified HPNE cell lines. **A**. Expression of oncogenic K-Ras and knockdown of Smad4 cooperate to induce EGFR, MMP9 and uPA expressions in HPNE and other K-Ras and Smad4 modified HPNE cells. **B**. The densitometry values were presented as the measurement of EGFR, MMP9 and uPA expression levels. The values were determined from multiple Western blots (n=3) analyses compared to the density of β-actin that was used as a loading control. The relative expression level of a particular protein was calculated by comparison to the density of the same protein in HPNE cells. Statistical significance value **p< 0.01 was calculated using student’s T-tests. **C**. Immunostaining followed by images from fluorescent microscopy showed the expression of EGFR, MMP9 and uPA in HPNE and genetically modified HPNE cells. Nuclei were visualized by staining with DAPI (blue). **D**. Samples (secreted proteins in serum free medium) were collected after 16 hours incubation of the cells in serum free medium. Gelatin and casein zymography analyses were performed to determine MMP9 and uPA activity.

 The parental cells, HPNE, or expanded isogenic clones of the three modified cell types (HPNE/ShSmad4; HPNE/K-Ras; and HPNE/K-Ras/ShSmad4) were analyzed for expression of EGFR, Smad4 and K-Ras by Western blot analysis. This analysis confirmed that expression of shRNA to Smad4 effectively knocked down the level of Smad4 expression in HPNE cells (HPNE/shSmad4) and in HPNE cells expressing oncogenic K-Ras (HPNE/K-Ras/ShSmad4) (Figure **1A**). In agreement with our previous finding, EGFR expression was increased in HPNE cells that express the oncogenic K-Ras transgene (HPNE/K-Ras and HPNE/K-Ras/ShSmad4), (Figure **1A**), [16]. Knockdown of Smad4 by itself was not sufficient to induce EGFR expression. However, knockdown of Smad4 in combination with expression of oncogenic Ras appears to further enhance the level of EGFR expression (Figure **1A**).

Our previous study showed that the increase in EGFR expression correlated with an increase in invasion [16]. To examine potential downstream molecular targets of EGFR signaling that might link EGFR activity with invasion, a comparison was done for expression of various proteolytic enzymes in the four cell line models. Initial studies suggest that MMP9 and uPA were up regulated in cells that express greater levels of EGFR. Western blot analyses showed that HPNE/K-Ras and HPNE/K-Ras/ShSmad4 had increased levels of MMP9 and uPA consistent with the increased levels of EGFR (Figure 1A.B). The expression and localization of EGFR, MMP9 and uPA were further determined by immunofluorescence and confocal microscopy (Figure **1C**). HPNE cells showed little EGFR, MMP9 or uPA expression; whereas, increasing cell surface/cytoplasmic expression of these molecules is seen in HPNE/K-Ras and HPNE/K-Ras/ShSmad4 cells (Figure **1C**). Moreover, the activities of MMP9 and uPA as determined by zymography showed similar increase to their expression levels. The HPNE cells showed almost no activity and an increase in activities of MMP9 and uPA were detected in HPNE/K-Ras and HPNE/K-Ras/ShSmad4 cells (Figure **1.D**). 

### Inhibitors of EGFR, NF-κB and PI3K block MMP9/uPA expression caused by oncogenic K-Ras and loss of Smad4

**Figure 2:**
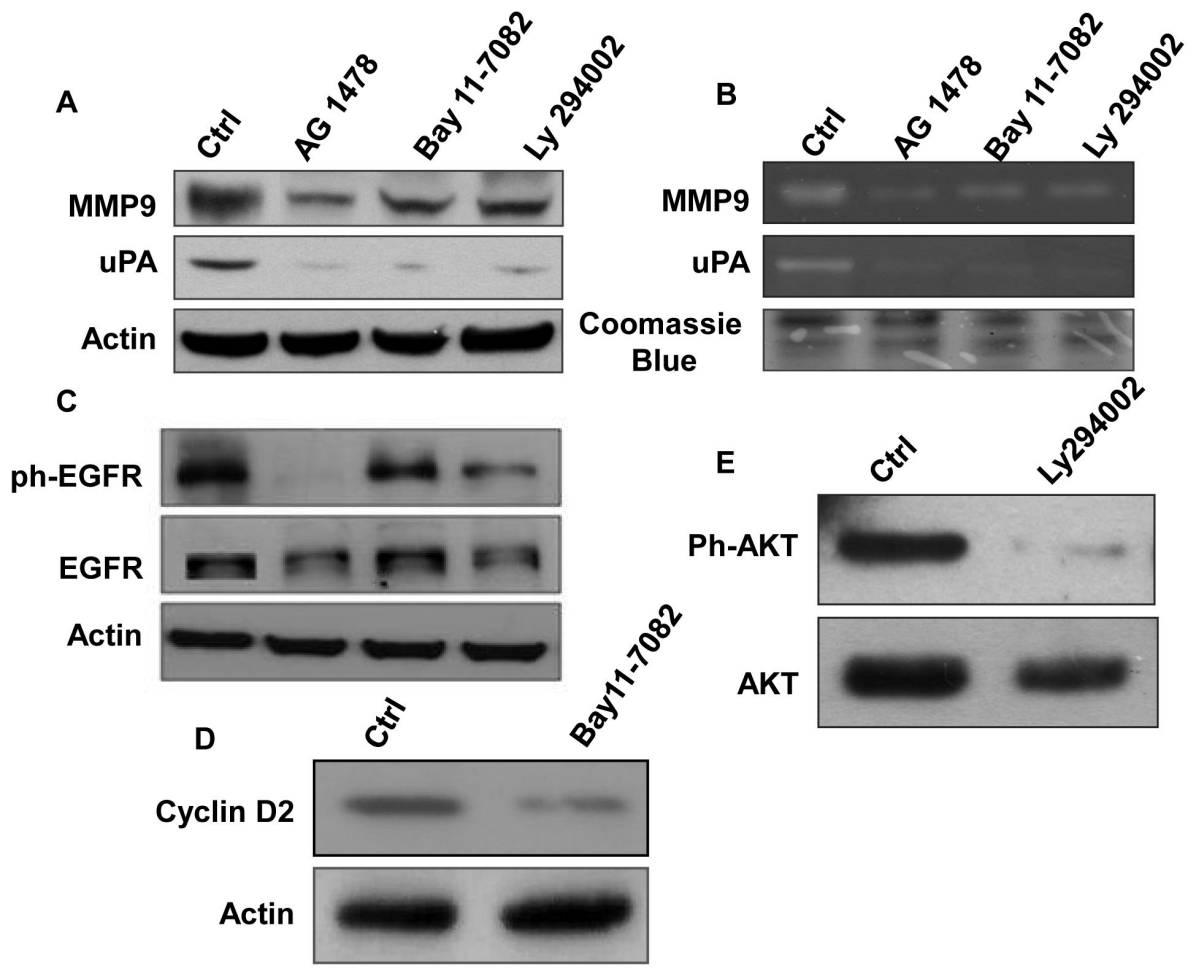
Expression level and activity of MMP9 and uPA was determined after treating HPNE/K-Ras/ShSmad4 cells with different inhibitors. **A**. Western blots analysis of MMP9 and uPA after treating cells with AG1478 (10 μM), Bay11-7082 (10 μM) or Ly294002 (10 μM). **B**. These inhibitors also blocked the secreted levels of MMP9 and uPA as determined by enzymatic activity in gelatin and casein zymography. **C**. Inhibition of the expression level of ph-EGFR by ph-EGFR, NF-κB and PI3K blockers, AG1478, Bay11-7082 and Ly294002 respectively at above concentrations. **D**. Western blot analysis was performed to monitor the cyclin D2 expression for the HPNE/K-Ras/ShSmad4 cells treated with Bay11-7082 to confirm the specific effect of Bay11-7082 [42] on NF-κB signaling. **E**. Western block showing that Ly294002 (10 μM) blocks AKT phosphorylation.

 A specific EGFR kinase inhibitor (AG1478) was used to determine whether there was a direct link between EGFR signaling and expression of MMP9 and uPA. Treatment of HPNE/K-Ras/ShSmad4 cells with AG1478 partially inhibited both MMP9 and uPA expression (Figure **2A**). This observation is consistent with previous reports indicating that EGFR signaling induces MMP9 expression in some types of cancer cells [29] [30]. The NF-κB transcriptional complex is reported to induce MMP-9 [31] and uPA [32] gene expression. In addition, EGF is reported to activate NF-κB in several cancers including human epidermal carcinoma cell lines, breast and prostate cancer cells [33] [34] [35]. Other studies indicated that NF-κB nuclear translocation promotes invasion of cancer cells [36-38] [39]. A recent study in prostate cancer suggests that MMP9 is induced through a PI3K/NF-κB pathway [40]. Moreover, EGFR signaling is widely known to activate both PI3K and NF-κB [33] [34,41]. To investigate the potential link between these pathways, HPNE/K-Ras/ShSmad4 cells were treated with inhibitors of NF-κB (Bay11-7082) and PI3K (Ly294002). As with the EGFR inhibitor, Bay11-7082 and Ly294002 both partially inhibited the expressions and activities of MMP9 and uPA (Figure **2.A, B**). As anticipated, treatment of cells with AG1478 completely inhibited the phosphorylation of EGFR (Figure **2.C**). Treatment of cells with Bay11-7082 had minimal effect on the expression or phosphorylation of EGFR; whereas, treatment with Ly294002 caused a decrease in expression and the level of phosphorylation of EGFR (Figure **2.C**). The expression of Cyclin D2, a known target of transcriptional activation by NF-κB, was inhibited by treatment with Bay11-7082 (Figure **2.D**). The specificity and activity of Ly294002 is supported by its capacity to inhibit the phosphorylation of AKT, a down stream target of PI3K (Figure **2** **. E**). Collectively, these studies support a role of EGFR, PI3K, and NF-κB for inducing the expression of MMP9 and uPA in HPNE/K-Ras/ShSmad4 cells.

### Expression of oncogenic K-Ras induces the nuclear translocation of NF-κB

**Figure 3:**
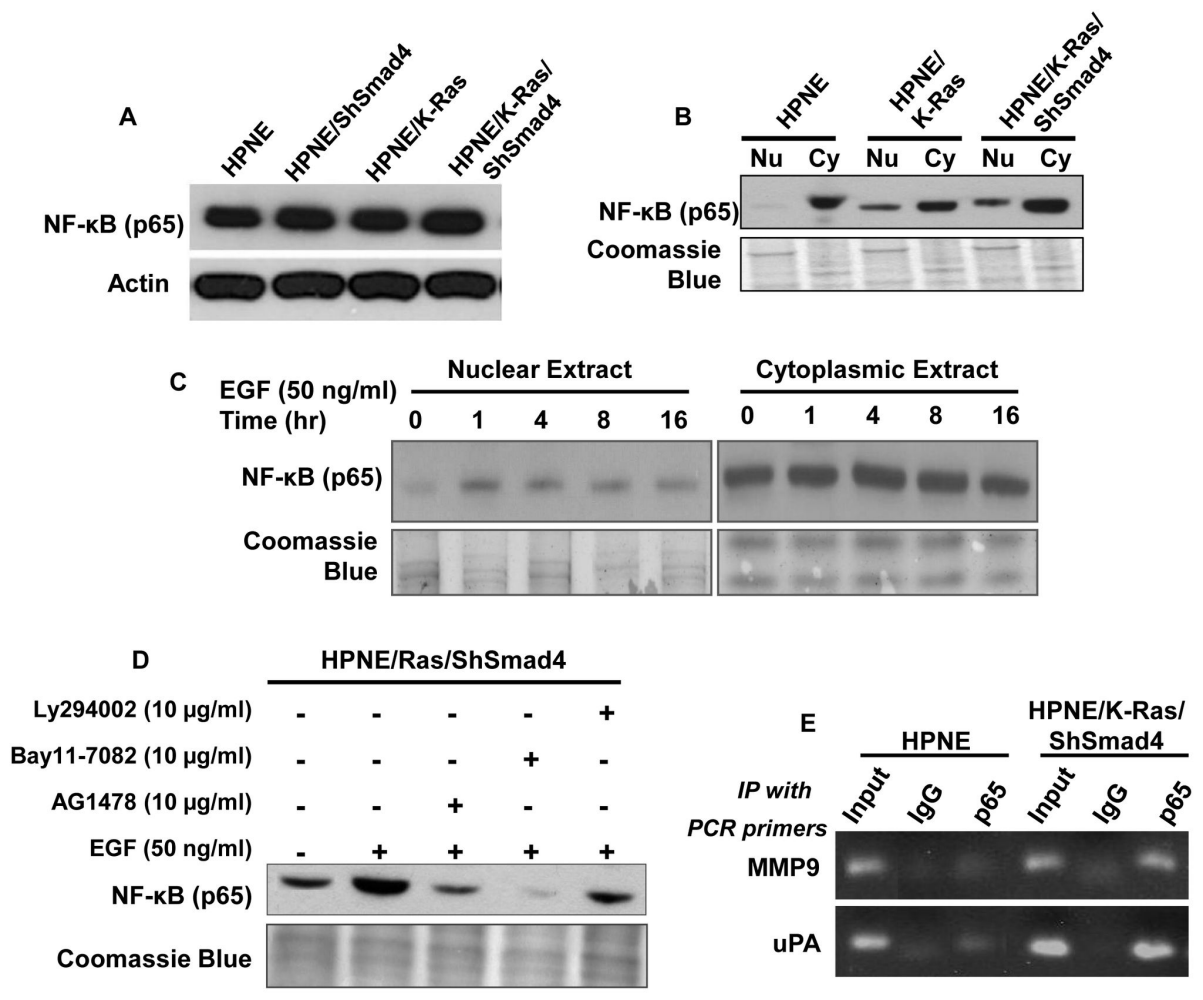
Nuclear translocation of NF-κB sub-unit. **A**. Western blot analysis for the NF-κB (p65) expression level in different genetically modified HPNE cells. **B**. Western blot analysis showing constitutive nuclear expression of p65 in cells expressing oncogenic K-Ras and loss of Smad4. **C**. Effect of EGF on nuclear translocation of NF-κB (p65). HPNE/K-Ras/ShSmad4 cells are treated with EGF (50 ng/ml) and harvested at indicated time points. Western blot analyses were performed with both nuclear and cytoplasmic extracts. A parallel Coomassie blue stained gel was presented as a loading control. **D**. Nuclear translocation of p65 is partially blocked by treatment of inhibitors described above in Figure 2. **E**. NF-B mediated regulation of uPA and MMP9. ChIP assay was performed to show the binding of NF-κB to the promoters of uPA and MMP9. The details for ChIP assay and PCR primers are described in material and methods section.

 The link between EGFR signaling with NF-κB activity was next investigated. RelA, the p65 subunit of NF-κB was expressed at similar levels in HPNE and the three HPNE modified cells (Figure **3A**). Next, the extent of cellular localization of RelA in cytoplasm or in nucleus was determined by Western blot analysis (Figure **3B**). RelA was predominately localized in the cytoplasm of HPNE cells; whereas, there was an abundant level of RelA found in the nucleus of HPNE/K-Ras and HPNE/K-Ras/ShSmad4 cells (Figure **3B**). Moreover, treating HPNE/K-Ras/ShSmad4 cells with exogenous EGF caused an increase in nuclear translocation of NF-κB by one hour that persisted up to 16 hours (Figure **3C**). Inhibitors of EGFR, PI3K, and NF-κB were used to examine the relationship among these three pathways. As anticipated AG1478, Ly294002 and Bay11-7082 blocked EGF mediated nuclear translocation of NF-κB. These results are consistent with that NF-κB is a downstream mediator of EGFR and induces the expression of MMP9 and uPA. Moreover, chromatin immunoprecipitation (ChIP) assays showed that RelA directly binds to the promoters of both MMP9 and uPA (Figure **3E**).

### Inhibitors of the EGFR/NF-κB axis inhibit invasion

**Figure 4:**
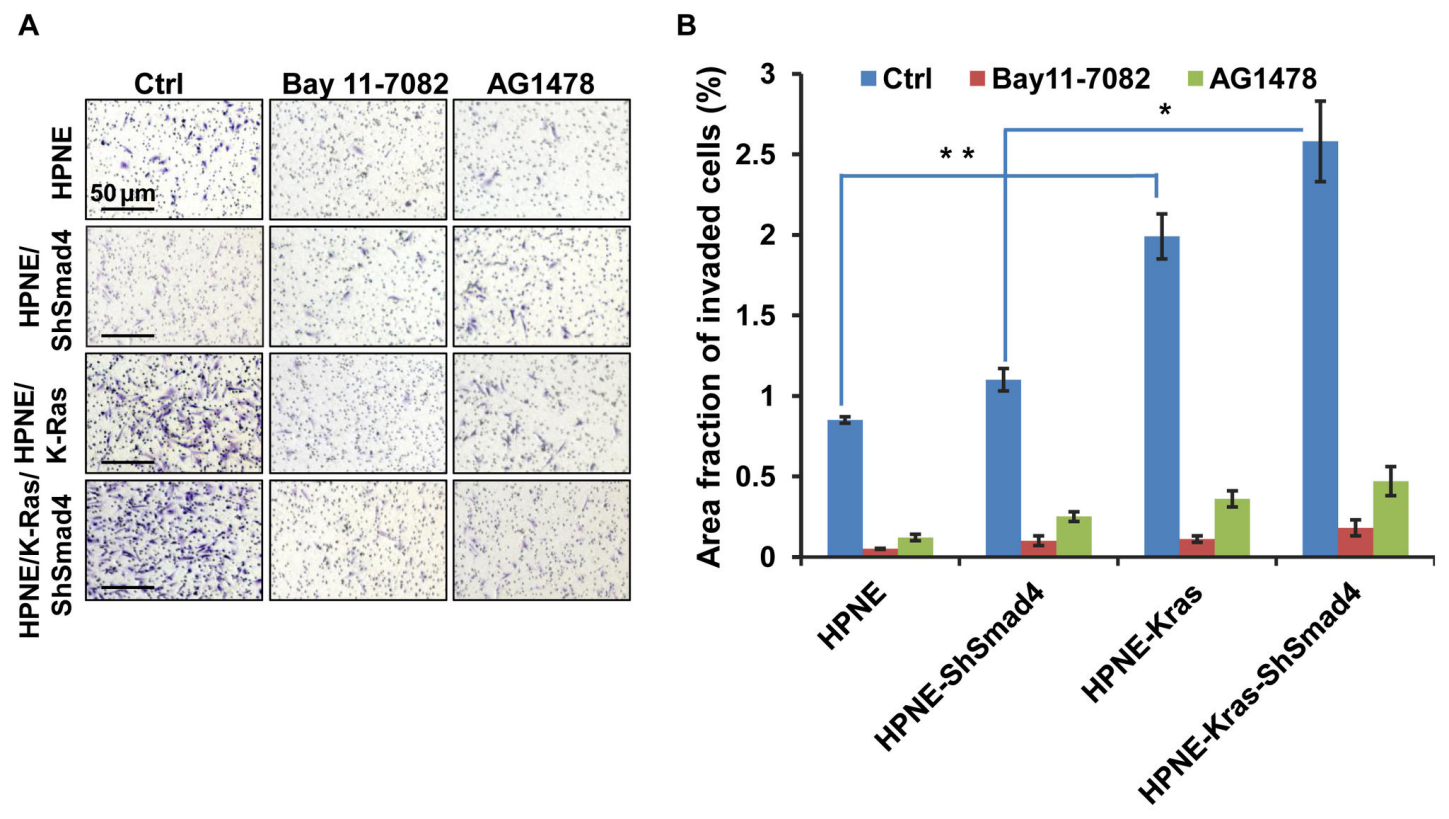
Loss of Smad4 and expression of oncogenic K-Ras induces invasion in HPNE cells. **A**. Images (10X magnification) were taken after 24 hours of seeding the cells in invasion chamber (BD Matrigel Matrix). **B**. Quantitative analyses were performed using *Image* *J* particle analysis program. Five individual images were taken from each chamber and then particle analysis was performed. Fraction of area occupied by the total particles (here indicates the invaded cells) are calculated by this analysis. Bars represent the standard deviation of five different images. Statistical significance of the data is presented as *p<0.05; **p< 0.01 using student’s T-test.

 In agreement to our previous findings, expression of oncogenic K-Ras induced invasion and invasion was further enhanced in K-Ras expressing cells by knockdown of Smad4 (Figure **4A**, left column). Moreover, treating cells with the EGFR inhibitor, AG1478 or the NF-κB inhibitor, Bay11-7082, prevented invasion (Figure **4A**, right columns). The quantitative assessment of invasion comparing the three cell models and the effects of the inhibitors on invasion is shown graphically in Figure 4B. 

**Figure 5:**
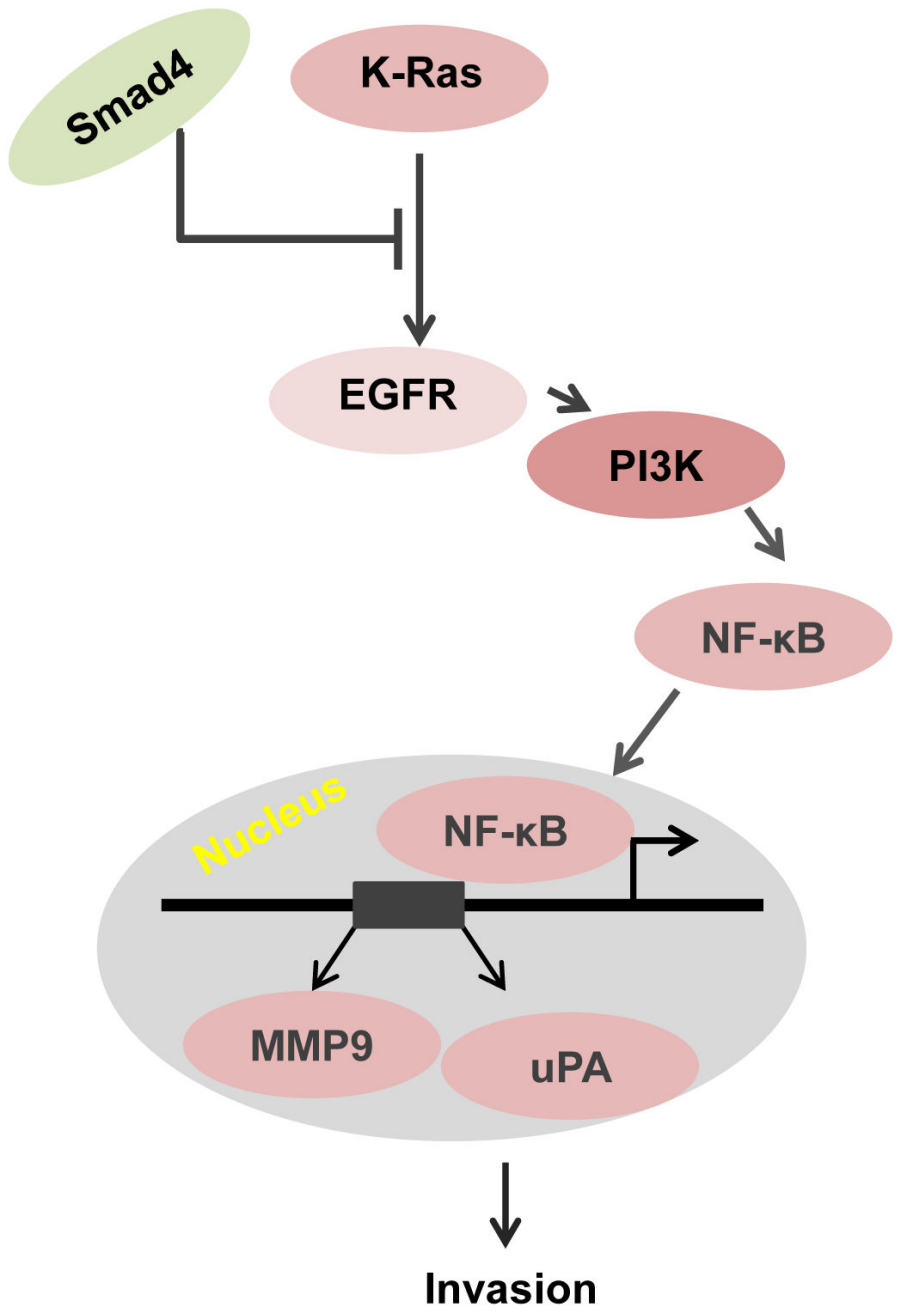
A working model is illustrating how oncogenic K-Ras and loss of Smad4 cooperate to cause an invasive phenotype. Oncogenic K-Ras signaling induces EGFR expression and K-Ras induced EGFR expression is normally suppressed by Smad4-dependent TGF-β signaling. Loss of Smad4 therefore leads to optimal up-regulation of K-ras induced EGFR expression. The increased expression and signaling by EGFR activates PI3K which induces nuclear translocation of NF-κB that in turn drives the expression of MMP9 and uPA.

 A schematic diagram suggesting the mechanism by which oncogenic Ras and loss of Smad4 cooperate to induce invasion is shown in Figure 5. These studies suggest that the oncogenic Ras induces the expression of EGFR and that Smad4-dependent TGF-β signaling would normally have a suppressive effect on Ras induced EGFR expression. However loss of Smad4 removes the suppressive effect resulting in further expression of EGFR. EGFR signaling and at least partially through PI3K induces the nuclear translocation of RelA. RelA then binds to MMP9 and uPA promoters driving transcription that in turn causes an increase in invasion. 

## Discussion

 The present studies sough to determine the mechanism by which expression of oncogenic K-Ras and combined with loss of Smad4 increases the cell migration and invasion of pancreas progenitor cells. Mutations of *K-Ras* and *DPC4* are two common alterations found in pancreatic tumors. Mutation of *DPC4* leads to loss of Smad4 expression or function and therefore loss of TGF-β tumor suppressor activity. EGFR is a member of the erbB/human epidermal growth factor receptor family of tyrosine kinases, which also includes erbB2/HER2, erbB3/HER3 and erbB4/HER4 [43] [44]. EGFR over-expression is reported in up to 90% of pancreatic tumors [45] [46]. Our previous data showed that induced expression of K-Ras triggers the expression of EGFR and erbB2 [16]. 

 Activating mutations of *K-*Ras leads to phosphorylation and activation of other kinases including ERKs and PI3K [47]. The activation of these downstream targets contributes to an increase in cell proliferation and survival [48]. Loss of TGF-β/Smad anti-proliferative and pro-apoptotic responses are common in cancer cells [49]. The loss of *DPC4*  by allelic deletion or intragenic mutation occurs in greater than 50% of PDAC as a relatively late event in tumor progression [7]. A study using genetic mouse models indicated that loss of Smad4 promotes progression of PDAC in the presence of activated K-Ras ^(G12D)^ [50]. These studies support a role of oncogenic K-Ras in establishment of pre-invasive pancreatic lesions and that a selective pressure to suppress Smad4-dependent signaling may contribute to PDAC progression. In this context, it is of interest that concomitant expression of oncogenic Ras is associated with up regulation of EGFR and erbB2 or other phosphotyrosine kinase receptors in pre-invasive pancreatic lesions [51]. Moreover, a study by Siveke et al [52] indicates, using a genetic mouse model, that up regulation of EGFR signaling is necessary for progression of pre-invasive pancreatic lesions to invasive disease. Our present studies indicate that oncogenic K-Ras and loss of canonical TGF-β signaling cooperate to induce the expression of EGFR and activation of MMP9 and uPA.

Different studies indicated that oncogenic K-Ras represses Smad signaling, suggesting that Smad-mediated tumor suppressor activity may be attenuated independent of mutation of *DPC4*[42,53]. A role for Smad signaling in suppressing oncogenic K-Ras induced tumorigenicity is supported by a study demonstrating that inactivation of Smad4 accelerated K-Ras (inducing G12D mutation) induced pancreatic cancer development and progression [50]. However, genetic studies in mice indicate that loss of Smad4 is not sufficient to cause the development of PDAC but contributes to tumor progression in the presence of additional oncogenic alterations [40] [54]. A separate study also indicated that patients with mutations of *DPC4* showed widespread metastatic disease compared to the more locally destructive disease of PDAC observed from patients with wild-type Smad4 [55]. These results provide a confirmation that *DPC4* (Smad4) is a PDAC tumor suppressor, functioning to block the progression of K-Ras (G12D)-initiated neoplasms by modulating both transcriptional and translational modifications of Ras. Our study supports and adds to these findings that loss of Smad4 enhances oncogenic K-Ras induced up-regulation of EGFR and contributes to the activation of NF-κB by translocation of p65 to the nucleus that in turn induces the expression of MMP9 and uPA and finally translates to invasion. 

 A previous report indicates that RelA, the p65 subunit of NF-κB, is constitutively activated in 67% (16 of 24) of pancreatic adenocarcinomas but not in normal pancreatic tissues [56]. Further, constitutive RelA activity was also detected in 9 of 11 human PDAC cell lines but not in non-tumorigenic Syrian golden hamster cell lines [56]. These results indicate that the NF-κB/RelA transcription factor is constitutively activated in many PDAC cells, which further up regulates the expression of urokinase-type plasminogen (uPA) activator [56,57]. Reports also indicate that EGFR regulates the constitutive activation of NF-κB in different cancer cells [52] [35] [58]. These data support a critical role of the EGFR/NF-κB pathway in cancer cell invasion and metastasis [35] [39]. One of the possible mechanisms is through increased nuclear translocation of NF-κB [59] [35]. In addition, other studies suggest a role of the Rho/ROCK/Ras pathway in tumor progression through the expression of NF-κB-dependent genes that are important for invasion [39] [60] [61] [62] [63]. Findings also support a contention that up-regulation of the Rho/ROCK pathway by LPA contributes to elevated proteolytic enzyme expression and subsequently cell invasion and metastasis [39]. Collectively these studies combined with the studies presented here suggest that oncogenic Ras causes NF-κB nuclear translocation that in turn induces MMP-9 and uPA expression. The study further suggests that EGF-induced nuclear translocation of NF-κB (p65 sub-unit) requires PI3K activity. 

The present study, for the first time, links oncogenic K-Ras and loss of Smad4 with an increased invasive phenotype by regulating proteolytic proteases through an EGFR/NF-κB pathway. Specifically, these data suggest that uPA and MMP-9 expression are regulated by a Ras dependent EGFR/NF-κB signaling cascade. More importantly, this study demonstrates that EGFR pathway is a critical intermediary that connects Ras to NF-κB nuclear translocation and subsequent proteolytic enzyme expression and invasion. 
